# Supplementary figures and images for: Effects of temperature and size class on the gut digesta microbiota of the sea urchin Tripneustes ventricosus
Source: PeerJ. 2024 Nov 28;12:e18298. doi: 10.7717/peerj.18298 (PMC11608566; doi:10.7717/peerj.18298)

**A.**

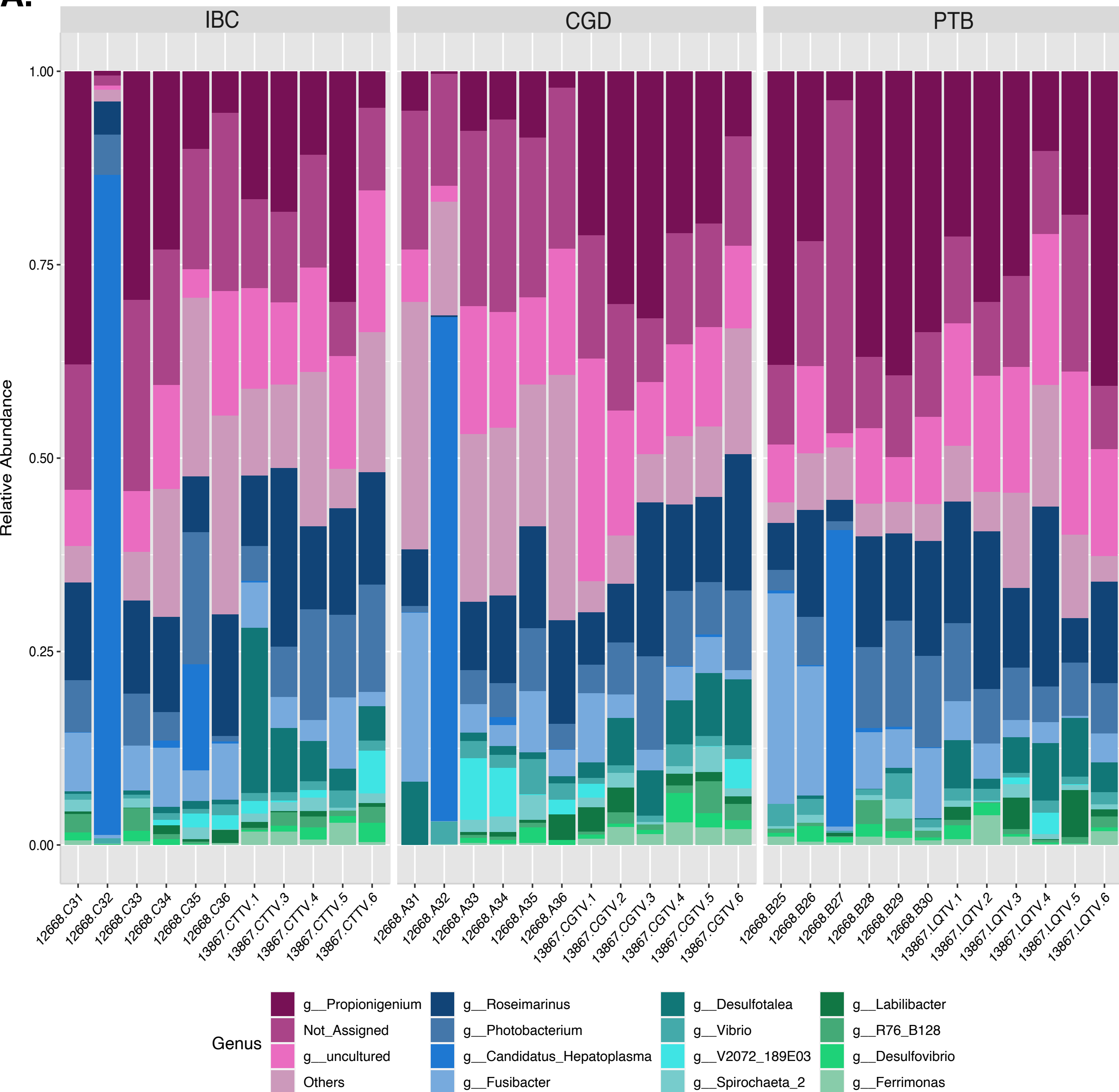

**B.**

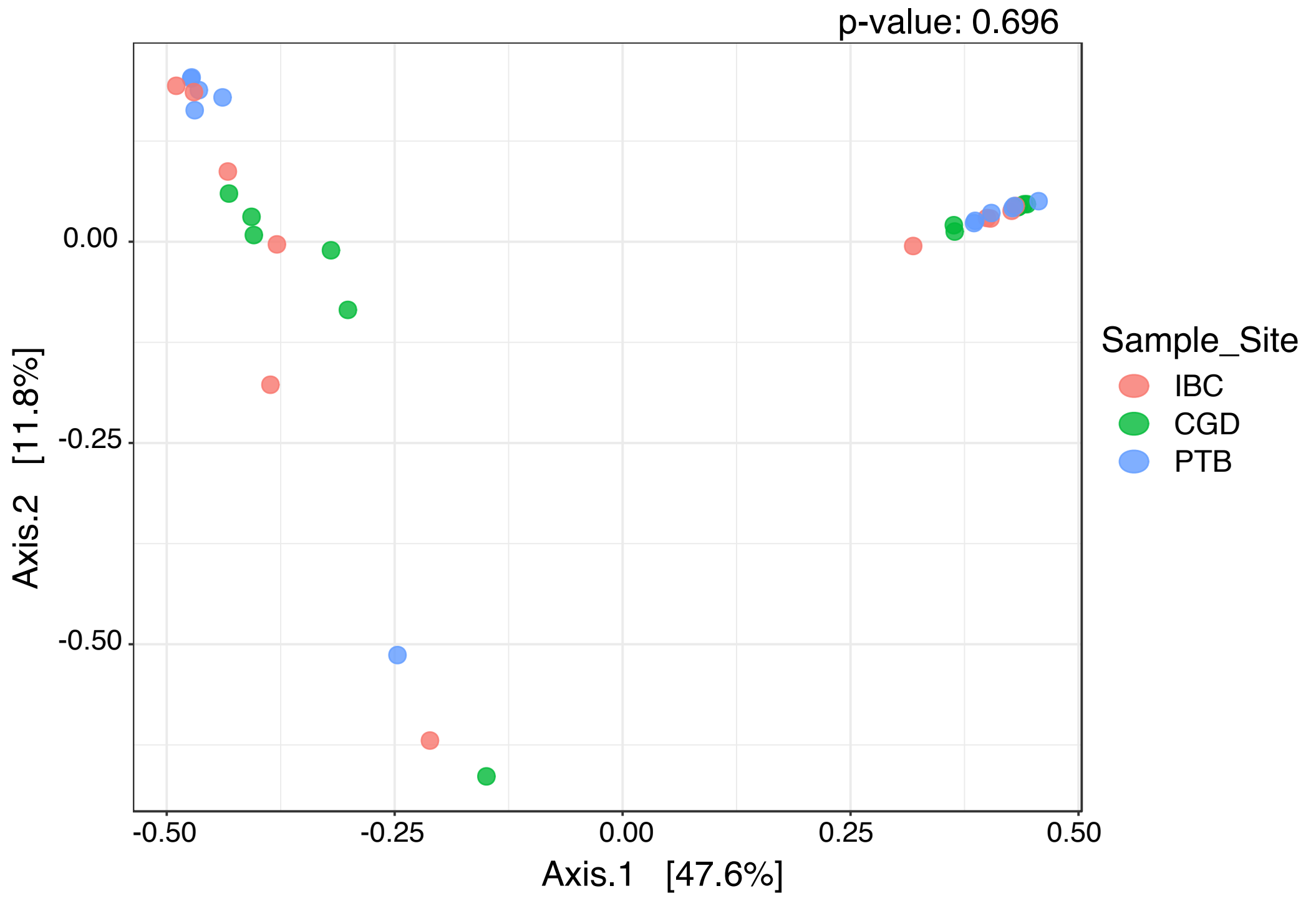

**C.**

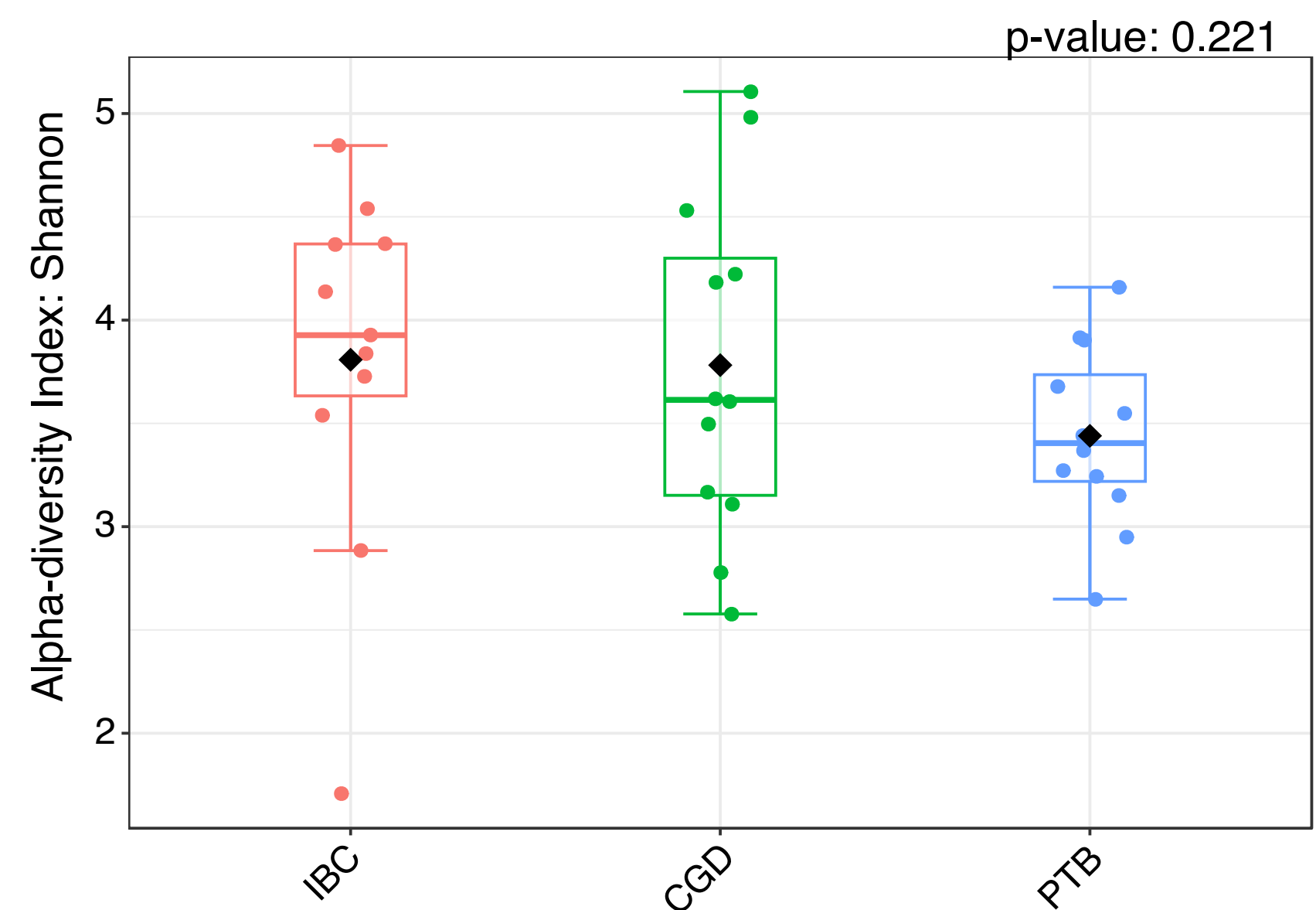

Supplement: Supplemental Information 1 — Taxonomic bar plot shows individual relative abundances per sample at genus levels (A). Beta diversityis representedin a 2D PCA plot using Bray-Curtis distances (B). Alpha diversity estimatesare visualizedby Shannon diversity boxplots (C). [file peerj-12-18298-s001.pdf]
