# Supplementary material for: Effects of temperature and size class on the gut digesta microbiota of the sea urchin Tripneustes ventricosus
Source: PeerJ. 2024 Nov 28;12:e18298. doi: 10.7717/peerj.18298 (PMC11608566; doi:10.7717/peerj.18298)
Supplement: Supplemental Information 2 — Asterisk indicates significant temporal differences (Kruskal-Wallis test, Chi2=12.5, pvalue=0.001). [file peerj-12-18298-s002.docx]

**Supplementary Table 1** - Abiotic parameters at three sites of Puerto Rico were Cerro Gordo (CGD), Isla de Cabra (ICB), and Punta Bandera (PBD). Asterisk indicates significant temporal differences (Kruskal-Wallis test, Chi^2^=12.5, p*_value_*=0.001).

| **Time** | **Site** | *** Water temperature (**ºC**)** | **Salinity (**o/oo**)** | **pH** |
| --- | --- | --- | --- | --- |
| February | CGD | 25.6 ± 0.00 | 33.3 ± 0.04 | 8.45 ± 0.05 |
|  | ICB | 26.1 ± 0.08 | 33.6 ± 0.05 | 8.41 ± 0.08 |
|  | PBD | 26.8 ± 0.07 | 33.8 ± 0.16 | 8.32 ± 0.05 |
| August | CGD | 29.9 ± 0.05 | 33.3 ± 0.08 | 8.45 ± 0.07 |
|  | ICB | 30.4± 0.01 | 33.6 ± 0.05 | 8.41 ± 0.16 |
|  | PBD | 30.8 ± 0.04 | 33.8 ± 0.08 | 8.32 ± 0.04 |
