# Supplementary material for: Effects of temperature and size class on the gut digesta microbiota of the sea urchin Tripneustes ventricosus
Source: PeerJ. 2024 Nov 28;12:e18298. doi: 10.7717/peerj.18298 (PMC11608566; doi:10.7717/peerj.18298)
Supplement: Supplemental Information 3 — Sites were Cerro Gordo (CGD), Isla de Cabra (ICB) and Punta Bandera (PBD). Size limit between small and large was established at 7.01 cm. Small were collected only in ICB. Asterisk represents significant differences between the two size classes within the same time-period (Mann-Whitney test, Z= 2.81, pvalue= 0.004). [file peerj-12-18298-s003.docx]

**Supplementary Table 2** - Average horizontal test diameter (mm) of sea urchin *Tripneustes ventricosus* in three sites of Puerto Rico. Sites were Cerro Gordo (CGD), Isla de Cabra (ICB) and Punta Bandera (PBD). Size limit between small and large was established at 7.01 cm. Small were collected only in ICB. Asterisk represents significant differences between the two size classes within the same time-period (Mann-Whitney test, Z= 2.81, p*_value_*= 0.004).

| **Time** | **Sites** | **Small (cm)** | **Large (cm)** |
| --- | --- | --- | --- |
| February | CGD | - | 10.0 ± 0.40 |
|  | ICB | * 6.0 ± 0.68 | * 9.6 ± 0.71 |
|  | PBD | - | 11.3 ± 0.93 |
| August | CGD | - | 8.7 ± 1.03 |
|  | ICB | - | 8.5 ± 1.04 |
|  | PBD | - | 10.6 ± 0.78 |
