# Supplementary material for: Effects of temperature and size class on the gut digesta microbiota of the sea urchin Tripneustes ventricosus
Source: PeerJ. 2024 Nov 28;12:e18298. doi: 10.7717/peerj.18298 (PMC11608566; doi:10.7717/peerj.18298)
Supplement: Supplemental Information 5 [file peerj-12-18298-s005.docx]

**Supplementary Table 4.** Comparative Statistical Outcomes of Beta and Alpha Diversity Indices, including PERMANOVA (strata), Shannon Diversity Index, Faith's Phylogenetic Diversity (PD) and Observed Features.

| **Comparison by factors** | **Analysis** | **p-value** | **q-value** |
| --- | --- | --- | --- |
| August (n=17) vs February (n=18) – Figure 1 | PERMANOVA (strata) | 0.001 | N/A |
|  | Shannon Index | 0.005564 | 0.005564 |
|  | Faith-pd | 0.390822 | 0.390822 |
|  | Observed Features | 0.002669 | 0.002669 |
| Large (n=17) vs Small (n=6) – Figure 3 | PERMANOVA (strata) | 0.789 | N/A |
|  | Shannon Index | 0.141482 | 0.141482 |
|  | Faith-pd | 0.441209 | 0.441209 |
|  | Observed Features | 0.528612 | 0.528612 |
